# Diversity, dissent, and fragmentation in the #MeToo movement: the role of collective and individual dimensions

**DOI:** 10.3389/fpsyg.2024.1290065

**Published:** 2024-06-26

**Authors:** Ana-Maria Bliuc, Tayla Hamilton, Daniela Muntele

**Affiliations:** ^1^Department of Psychology, School of Humanities, Social Sciences, and Law, University of Dundee, Dundee, United Kingdom; ^2^School of Psychology, Western Sydney University, Penrith, NSW, Australia; ^3^Department of Psychology, Alexandru Ioan Cuza University, Iași, Romania

**Keywords:** social identity, social movements, dissent, collective action, #MeToo movement, intragroup processes

## Abstract

**Introduction:**

In this research, we examine how intragroup fragmentation, which is the division of a group into smaller subgroups, interacts with different forms of action against gender inequality. We focused on two types of action: actions that promote social change and actions that encourage retribution.

**Methods:**

We investigated these processes within the #MeToo social movement using data collected in Australia (*N* = 363) and Romania (*N* = 135). In both samples, we measured antecedents of ’group consciousness’ (previous experience with discrimination, empathic concern, and perspective taking) and its indicators (social identification, perceived group efficacy beliefs, and group emotions such as anger and contempt). As indicators of intragroup fragmentation, we measured endorsement of different categories of group behaviors such as pro-social change action versus pro-retribution action. To assess the predictive power of motivations for joining the movement (antecedents of group consciousness) and of group consciousness for either pro-social or retributive actions, we tested several structural equation models (SEMs).

**Results:**

Our results indicate that the motivations for joining such social movements were more complex than anticipated, with perspective-taking emerging as a significant differentiator. Our analyses further show that different dimensions of group consciousness could predict support for either pro-social or retributive actions.

**Discussion:**

These findings highlight the complexity of the intragroup processes in newly emerging, modern social movements such as #MeToo. Our findings have implications for the study of membership dynamics in social movements and suggest that strategies to mobilise support should be tailored to these complexities. Overall, this research contributes to the current understanding of intragroup dynamics in contemporary social movements, thereby providing insights that could inform both grassroots mobilisation strategies and policy interventions aiming to increase gender equality.

## Introduction

Society is often deeply divided across fault lines caused by dissent on important social issues such as global warming, immigration, gun control, and gender equality (Abramowitz and Saunders, 2008; Ramos et al., 2015). How we position ourselves in relation to such issues creates ideologically opposed camps – that is, psychological groups based on contrastive collective narratives about important aspects of social reality (Bliuc et al., 2020, 2021). Ideologically opposed camps are underpinned by a *group consciousness* (Duncan, 2012) conducive to collective action toward group goals in line with the camp’s collective narrative (Bliuc et al., 2021; Bliuc and Chidley, 2022).

When groups hold divergent beliefs or have conflicting interests, dissent and intergroup conflict can arise. This can be a powerful force for disrupting the status quo and ultimately leading to significant social change that makes society more just and cohesive (Klandermans, 1997; van Zomeren et al., 2008; Drury et al., 2012). Although intergroup conflict may not be inherently positive, it is often the catalyst for positive social change. For example, the strong ideological conflict over racial equality during the Civil Rights Movement in the US ultimately achieved social progress (Bliuc et al., 2007, 2015, 2021; McGarty et al., 2009). Initially sparked by dissent and Black activism in response to systemic injustices and human rights violations, the movement ultimately evolved into a fully-fledged movement that transcended racial self-categorization, so that support for the movement’s cause and principles became the basis of self-categorization. As such, while intergroup conflict can be a disruptive force, it also has the potential to drive important social change and transformation.

While at the level of society, dissent can function as a driver of intergroup conflict and division into opposed camps (Bliuc et al., 2023), within camps, it can produce *intragroup fragmentation*. That is, dissent that emerges within a group may lead to intragroup fragmentation and the formation of splinter factions, that, when the bases for dissent are about fundamental issues of group identity and therefore make compromise impossible, they would eventually break away from the parent group (Sani and Todman, 2002; Sani, 2008; Bliuc et al., 2015, 2021). Dissent can occur as an unintended result of social movement diversification (Cunningham et al., 2017; Wang et al., 2019), when social movements use different strategies, usually non-violent to achieve their aims (such as protests, blockades, strikes, social media campaigns, etc.), and there are disagreements within the movement about these strategies – such as for example in terms of their efficacy.

In our research, we focus on the potential of dissent as a driver of intragroup fragmentation. Specifically, we examine dissent in the form of support for different types of group behaviors to achieve the group’s goals, and which in turn can fragment and weaken l groups and social movements. We propose that intragroup fragmentation can be understood as commitment to different group goals and as such be reflected in the group members’ endorsement of different types of group behaviors that are aligned with the group norms in varying degrees [see Thomas and Louis (2014), Jimenez-Moya et al. (2015), and Saab et al. (2016)]. For instance, in groups based on narratives about injustice, their goals are often about achieving profound changes in the conditions that enabled the injustice to occur (i.e., *social change-oriented goals*), as for example in the US civil rights movement. They can co-exist with goals of remedying the injustice by punishing those responsible (i.e., *retribution-oriented goals*). From this categorization, group behaviors can be seen as: (a) behaviors aimed to achieve social change —remedying the injustice by changing the society, and (b) behaviors aimed at punishing those responsible —achieving retribution by punishing the perpetrators of injustice. Support for these types of behaviors is not mutually exclusive, in the sense that group members could endorse both types of behaviors, but we would expect that some members will tend to endorse one type over the over.

Here, our investigation of intragroup fragmentation is conducted in the context of progressive, ideological camp based on narratives about gender injustice and consensus about addressing this injustice, that is, the #MeToo social movement. The #MeToo movement is a global social movement against sexual violence and harassment (Shugerman, 2017; Xiong et al., 2019). Founded in 2006 by Tarana Burke, it gained widespread momentum in 2017 following allegations against Harvey Weinstein (Kantor and Twohey, 2017; Jagsi, 2018; Mendes et al., 2018). The movement is a perfect example of a group based on consensus, emerging from shared beliefs about gender inequality and actions to address it.

**Figure 1:**
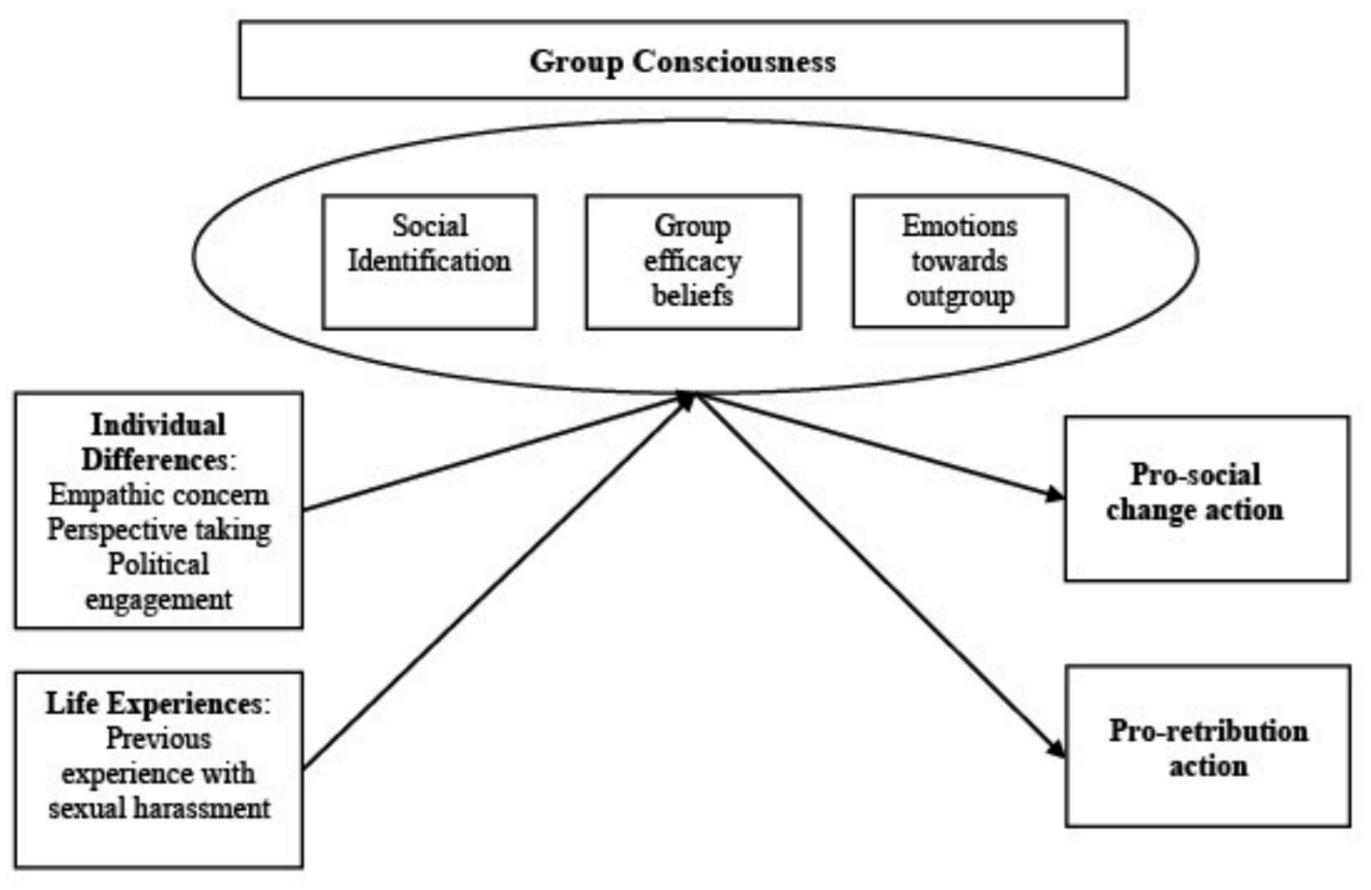
Theoretical model illustrating the antecedents and outcomes of group consciousness.

As a basis of fragmentation in this movement, we investigate support for different categories of behaviors – i.e., behaviors consistent with a social change-oriented pathway (pro-social change action) and behaviors consistent with a retribution-oriented pathway (pro-retribution action). Based on contemporary models of collective action (Duncan, 2012; Thomas et al., 2018) support for these different types of behaviors are driven by different predictors (i.e., dimensions of group consciousness). The theoretical model that this research is based on is presented below in Figure 1.

### Dimensions of group consciousness

Collective action is thought to stem from group consciousness, which combines group identification, efficacy beliefs, and perceptions of injustice (Duncan, 1999, 2012; Bliuc et al., 2015; Thomas et al., 2016). This encompasses social psychological variables related to group identification, analysis of a group’s societal position, and a collective orientation toward balancing power between groups (Duncan, 2012). In other words, individuals are more likely to participate in collective action, including polarized action when they identify with the group, have strong emotional reactions to perceived injustice (anger or contempt), and believe in the group’s ability to achieve goals (van Breen et al., 2017).

We propose that group members’ preference for different types of behaviors (pro-social change action versus pro-retribution action) should be driven by different experiences of activism (group consciousness as captured by levels of group identification, perceived group efficacy), and perceptions of ingroup injustice (as emotions toward outgroup members). Next, we discuss in more detail the three components of group consciousness —identification with the group, group efficacy beliefs, and perceptions of ingroup injustice.

#### Group identification

Group identification is key in understanding the experience of group belonging as it incorporates beliefs about who we are, what we stand for, and what we can do to achieve our goals (Livingstone and Haslam, 2008). Consensualization of identity content occurs through interaction between members (Postmes et al., 2005a,b; Thomas et al., 2010), but in cross-cultural movements like #MeToo, communication is often conducted online via written text – a relatively ‘lean’ communication channel (Daft and Weick, 1984; Daft and Lengel, 1986) – which in turn can increase ambiguity. Thus, while #MeToo supporters are brought together by consensus on the issue of addressing gender inequality and stopping the sexual exploitation of women, it is plausible that understandings of what it means to be a supporter and what is expected from them as supporters may differ between supporters within the movement. These differences are likely to be reflected in different levels of identification with the movement (as well as the other dimensions of group consciousness).

#### Perceptions of group efficacy

Perceptions of group efficacy, that is, the belief that the ingroup is capable to achieve its goals have been shown to = explain participation both normative collective action and non-normative (radical) collective action. In particular, when peaceful action is not seen as efficacious by group members (i.e., there are low levels of perceived group efficacy), a ‘nothing to lose’ strategy underpinning support for violent collective action can be employed (Saab et al., 2016). When group members legitimize hostile or other forms of illicit behaviors as effective in advancing their collective goals, it can lead to increased support for and adoption of radical behaviors (Tausch et al., 2011; Thomas et al., 2014; Becker and Tausch, 2015; Jimenez-Moya et al., 2015; Saab et al., 2016). Put differently, this research suggests that support for non-normative group behaviors is underpinned by dissent on what is an effective strategy to achieve the group’s goals.

#### Perceived injustice toward ingroup

Much of the research on collective action uses negative emotions toward the outgroup as an indicator of perceived injustice. Experiencing negative emotions such as anger, outrage and contempt as a group member was shown to drive different types of behavior (van Zomeren et al., 2004). For example, research on the social construction of emotion views anger as a productive emotion which functions to correct misconduct and uphold accepted standards of behavior (Averill, 1983; Weber, 2004). Social appraisals of group-based events resulting in anger have been associated with moderate collective action (Mackie et al., 2000; Walker and Smith, 2002; van Zomeren et al., 2004; Duncan, 2012). On the contrary, contempt has been associated with a lack of reconciliation intent, dehumanization (Fischer and Roseman, 2007; Esses et al., 2008), and more extreme forms of group behavior (Tausch et al., 2011; Feddes et al., 2012; Becker and Tausch, 2015). In particular, Tausch et al. (2011) found that anger predicts normative collective action in two studies (although not in the context of Indian Muslims’ support for actions related to ingroup disadvantage, where perceptions of injustice were found to predict normative action). Contempt, on the other hand, was found to positively predict intentions to engage in non-normative action in multiple contexts, including German student protests, political violence among Indian Muslims, and support for violence against military and civilian targets among British Muslims.

These differences in emotions toward the outgroup within the same group are important not only because they are likely to underpin different types of group behaviors, but also because they suggest that there may be different drivers to support for the movement that underpin these differences. They point out to the connections between the emotional content of group consciousness and individual factors such as the capacity for empathy. One line of research supporting this point is that on dehumanization as connected to a lack of empathy (Bandura, 1990; Castano, 2012; Leidner et al., 2013). It is likely that other individual factors are involved not only in the expression of group-based emotions, but also in affecting the other dimensions of group consciousness. We discuss next some potential individual factors that shape group consciousness and in turn a preference for different types of group behaviors.

### Antecedents of group consciousness

The antecedents of group consciousness have been conceptualized as life experiences as well as other individual factors, such as experiences of discrimination for example (Duncan, 2012; Bliuc et al., 2015). Similar to other progressive social movements that appeal to various segments of society, #MeToo brings together both people directly affected by gender discrimination and allies, that is, people who might have never experienced gender discrimination, but are driven by a sense of solidarity with the victims. Both these categories of group members may share a subjective sense of identification with the movement’s cause, with the specific collective narrative that underpins its cause, and with others committed to the cause (Subašić et al., 2008; Saab et al., 2015). They would also develop group consciousness, similarly, fuelled by group efficacy and perceptions of injustice. However, despite these commonalities, there is also a lot of diversity within those identifying as #MeToo supporters (the distinction between victims and allies being only one of many others), and therefore it is highly plausible that the experience of group belonging (as captured by group consciousness) is shaped by differences in individual factors such as different life experiences, interest in society and politics (political engagement), and individual differences such as levels of empathy.

#### Life experiences

We propose that supporters of #MeToo experience group membership differently based on their motives for joining the movement, including commitment to gender equality and personal experiences with discrimination. Previous research shows that experiencing discrimination, specifically sexual harassment, is a key predictor of activism, likely shaping the subjective experience of being a supporter of the movement (Duncan, 1999; Liss et al., 2004; Friedman and Leaper, 2010). This experience can lead to higher psychological and emotional investment in the group, as captured by group identification and group-based emotions (Duncan, 2012).

#### Political engagement

Political engagement is a higher order concept incorporating attitudes and behaviors indicating attention, cognitive engagement, and interest in politics and governance (Prior and Boucher, 2018). Levels of political engagement were found to be affected by individual factors such as gender, age in young people, and economic inequality (Verba et al., 1997; Solt, 2008; Levy and Akiva, 2019). Therefore, this construct can be used as a proxy for several individual variables (including socio-economic status and a cognitive openness/curiosity toward politics).

#### Empathy

We propose that individual differences in the capacity for empathy are key in shaping the subjective experience of activism for supporters. These factors are important because they might explain differences in supporters’ emotions and, respectively, interpretations of group norms (in relation to behavioral responses to members of an opposing outgroup, Levin et al., 2016). Empathy is broadly defined as the reaction one has to the experiences of another (Davis et al., 1999). It relates to social cognition and comprises of two main elements: an emotional aspect (empathic concern) and a cognitive aspect (perspective taking) – the ability to grasp the thoughts and emotions of other human beings (Gagen et al., 2015). This functional difference links empathic capacity with the emotional responses associated to the experience of collective support. We argue that differences in empathy can help us understand emotional responses such as increased anger versus contempt (Vitaglione and Barnett, 2003). Specifically, empathy is a critical factor in understanding why people who have never experienced sexual discrimination or harassment themselves may still be committed to supporting the #MeToo movement (Batson et al., 2002; Batson and Ahmad, 2009; Bongiorno et al., 2020). In this case, empathy can help explain the altruistic drivers of collective support and levels of commitment to the movement.

### Intragroup fragmentation as support for different types of group behaviors

Social identity theory (Tajfel and Turner, 1979; Turner et al., 1987) suggests that group members expect agreement on important aspects of their social identity, such as core beliefs, values, aims, and norms. When this agreement is compromised, the group’s internal cohesion may suffer. Research on schism shows that if members of a subgroup believe the group identity has been undermined, they may consider leaving the group, resulting in a schism (Sani and Todman, 2002). This can lead to subgroups leaving the parent group to form a splinter faction or joining a different group (Sani and Reicher, 1999; Sani and Todman, 2002).

Sani and Todman (2002) studied the potential for group fragmentation by examining intentions to leave the Church of England’s clergy in response to debates about the ordination of women as priests. They found that members of a faction may want to split from the main group if they believe that a new norm challenges a fundamental aspect of group identity. We build on this idea by proposing that intragroup fragmentation can occur when group members have different views on the best way to achieve the group’s goals, even if they do not openly disagree on core issues of group identity. This can create a platform for dissent that may lead to ideological fragmentation and schism. It is not necessary for group members to see certain behaviors as not representing the group, but rather they may perceive some behaviors as more effective than others.

Here, we aim to investigate the factors contributing to group fragmentation and ultimately schism, focusing on the role of intragroup dissent and support for different types of group behavior. Our categorization of pro-social change versus pro-retribution behaviors can be seen as broadly aligned to the categorization into normative and, respectively, non-normative group behaviors. Non-normative behaviors, such as sabotage and physical attacks, can indicate a departure from core group values and norms, potentially leading to intragroup fragmentation (Thomas and Louis, 2014; Saab et al., 2016). Furthermore, research suggests that group members are more likely to engage in non-normative behaviors when they perceptions of group efficacy are low and group members feel contempt rather than anger at the outgroup (Tausch et al., 2011). Additionally, group identification plays a crucial role in determining which types of behaviors are preferred, with low group identifiers being more likely to endorse non-normative behaviors (Jimenez-Moya et al., 2015). These findings highlight that disagreements about the means to achieve group goals can lead to low group identification and eventual group fragmentation.

## Present research

In applying these insights to the understanding of ingroup fragmentation in the #MeToo social movement, we propose that within the movement, we should be able to identity at least two subgroups of supporters that can be differentiated on the basis of their endorsement of either pro-social change behaviors or pro-retribution behaviors. We expect that people supporting pro-social change behaviors over pro-retribution behaviors will differ from the ones supporting pro-retribution over pro-social change behaviors in the variables capturing group consciousness. That is, we expect that these subgroups would differ in terms of their levels of group identification, emotions toward the outgroup (anger versus contempt), and perceptions of group efficacy. Specifically, those endorsing pro-social change group behaviors would be higher in identification, anger, and perceptions of group efficacy than those endorsing pro-retribution group behaviors, who in turn will be higher in contempt. Two studies were conducted to test our hypotheses:

1. *Collective dimensions of intragroup fragmentation:* Within the supporters of the #MeToo movement, those endorsing pro-social change action over pro-retribution action will differ in their group consciousness dimensions from those endorsing pro-retribution action over pro-social change action; that is, pro-social change action supporters are expected to be higher in group identification, anger, and group efficacy compared to pro-retribution action supporters, who in turn are expected to be higher in contempt (but lower on all the other collective dimensions).
2. *Individual dimensions of intragroup fragmentation*: We predict that supporters endorsing pro-social change action will differ in their motivations for joining the movement (antecedents of group consciousness) from those endorsing pro-retribution action; that is, we expect that the first group will be higher in political engagement and lower in previous experiences of sexual harassment than the second group. Empathy on the other hand, should not vary according to whether supporters endorse pro-social change or pro-retribution action.
3. *The pathways to addressing gender inequality*: we expect that support for pro-social action and support for pro-retribution action will be predicted by different dimensions underpinning group consciousness. That is, pro-social change action should be predicted by a latent variable (“Group Consciousness”) as indicated by identification, group efficacy, and anger, while pro-retribution action would be predicted by a latent variable indicated by identification, group efficacy, and contempt.

The studies were conducted in two different cultural contexts: Australia and Romania. By using populations from these two countries which also differ significantly in their rankings in the 2022 Global Gender Gap Index (43rd and, respectively, 90th), our objective was to ensure that our findings are not based on a standard WEIRD population only. The population drawn from Romania is also interesting because Romania is one of the Eastern European countries where traditional gender roles are still dominant (Inglehart et al., 2014) and is one of the three countries with the lowest scores on gender equality in Europe (Gender Equality Index, 2022).

### Methods

Both studies were conducted online. In Study 1, conducted in Australia, we measured antecedents of group consciousness (previous experience with discrimination, empathic concern, and perspective taking), indicators of group consciousness (including social identification, group efficacy beliefs, and group emotions such as anger and contempt), and endorsement of different categories of group behaviors (pro-social change and pro-retribution action) in the context of the #MeToo movement. The participants completed an online survey via Amazon Mechanical Turk, with informed consent being obtained from participants prior to the commencement of the survey. After reading a short introduction about the gender equality debate and #MeToo movement, participants classified themselves as either a #MeToo supporter or as a non-supporter depending on the category most representative of their ideological stance.

In Study 2 a similar procedure was used. However, the data was collected from a population from a non-Western culture, that is, the online survey was distributed via Google Forms to a general population from Romania. The questionnaire was translated into Romanian and back translated by two of the authors who are native Romanian speakers. The same ethics protocol as in Study 1 with participants indicating consent for their participation in the research was followed. In addition to the measures used in Study 1, in Study 2 we used more precise measures for several variables. That is, we included measures of emotions toward potential perpetrators of injustice as an indicator of group consciousness (rather than just emotions toward the outgroup) and measures of both direct and indirect experiences of sexual harassment.

### Participants

Participants had read a brief introduction to issues surrounding gender equality and the #MeToo movement followed by a self-categorization task (asking participants to indicate if they identify as a supporter of the #MeToo movement). In Study 1, we analyzed data collected from the participants who self-identified as supporters of the #MeToo movement (*N* = 363). The sample included both women (*N* = 196) and men (*N* = 167) with ages ranging from 19 to 86 (*M* = 36.72, *SD* = 11.79).

In Study 2, data from a total number of 135 Romanian participants was collected. From these, we excluded 9 participants who did not self-identify as supporters of the #MeToo movement. The final sample included 126 self-identified #MeToo supporters, including 98 women and 28 men with ages ranging from 18 to 62 (*M* = 30.72, *SD* = 12.18).

### Measures

Unless otherwise specified, for both studies, all variables were measured using a 7-point Likert scale ranging from 1 = *strongly disagree*, to 7 = *strongly agree*.

#### Endorsement of different categories of group behaviors

Support for group behaviors was measured using items adapted from Bliuc et al. (2015). In Study 1, an exploratory factor analysis (using promax oblique rotation, Bartlett’s *χ*^2^ = 4975.18, *p* < 0.001) revealed that the items loaded on 2 factors underpinning a preference for group behaviors aimed toward achieving social change (factor 1 loading sum squared of 5.33; 9 items, e.g., signing a petition, attending a peaceful political rally, voting for a candidate in the next election whom promotes gender equality), and, respectively, a preference for behaviors aimed toward punishing potential perpetrators (factor 2 loading sum squared of 2.78; 5 items, e.g., public humiliation of, or undermining the business of an alleged sexual offender, vandalizing property). Thus, we computed a pro-social change subscale (*α* = 0.930) and, respectively, a pro-retribution sub-scale (*α* = 0.832). For Study 2, the same measure was used. Confirmatory factor analysis supported the same two-factor structure as in Study 1 (Bartlett’s *χ*^2^ = 1080.38, *p* < 0.001), so we computed one sub-scale of 10 items measuring preference for group behaviors aimed toward achieving social change (pro-social change action, *α* = 0.924), and a second subscale of 5 items measuring preference for behaviors aimed toward punishing potential perpetrators (pro-retribution action, *α* = 0.762).

#### Social identification

In both studies, the participants responded to a 14 items scale adapted from an existing measure of social identification (Leach et al., 2008). Participants responded to items such as “I feel a bond with other people who support the #MeToo movement” and “I feel committed to #MeToo supporters.” Items were averaged to yield a composite score for social identification with #MeToo supporters (*α* = 0.959 for Study 1 and *α* = 0.951 for Study 2).

#### Perceptions of group efficacy

Items measuring perceptions of group efficacy were adapted from van Zomeren et al. (2008) to the context of the #MeToo movement for both studies. Participants responded to three items (*α* = 0.913 for Study 1 and *α* = 0.967 for Study 2) regarding their beliefs in movement’s capacity to make effective change (e.g., “I feel that together #MeToo supporters will be able to improve the outcomes for women in our country”; “I feel that #MeToo supporters will be successful in their aims”).

#### Emotions toward outgroup

In Study 1, negative emotions toward outgroup were measured using 7 items adapted from previous studies (Bliuc et al., 2015). Exploratory factory analysis (using a promax oblique rotation, Bartlett’s *χ*^2^ = 1785.48, *p* < 0.001) indicated that the items loaded on two factors underpinning the dimensions of *anger* (factor 1 loading sum squared of 2.69; i.e., feeling angry, disgusted, and outraged; *α* = 0.939) and *contempt* (factor 2 loading sum squared of 1.09; i.e., feeling hateful and amused). Similarly, in Study 2, we created composite scores for anger (4 items, *α* = 0.937) and contempt (3 items, *α* = 0.865). Confirmatory factor analysis supported the two-factor solution from Study 1 (Bartlett’s *χ*^2^ = 721.736, *p* < 0.001).

#### Emotions toward potential perpetrators of injustice (Study 2 only)

The same items used to measure emotions toward outgroup were used to capture emotions toward perpetrators by changing the target of emotions in the question. That is, rather than asking participants to indicate their emotions toward people who hold an opposite position toward the #MeToo movement than themselves, we asked participants about their emotions toward potential perpetrators of sexual harassment and gender discrimination. The same as for emotions toward the (ideological) outgroup, confirmatory factor analysis supported a two-factor solution (Bartlett’s *χ*^2^ = 780.183, *p* < 0.001), so a subscale for anger (4 items, *α* = 0.953) and, respectively, one for contempt (3 items, *α* = 0.830) were created.

#### Political engagement

Four items assessing importance, commitment, and interest in politics and general levels of activism were used in both studies (e.g., “How important are your political beliefs to you personally?”; “How interested are you in politics and political issues?”; 4 items, *α* = 0.90 for Study 1 and *α* = 0.89 for Study 2). One item was used to measure political orientation (i.e., liberal or conservative).

#### Previous experience with sexual harassment

Measures of previous experience with sexual harassment were taken using items from the *Unwanted Explicit Sexual Advances* subscale (3 items, e.g., “How often has someone made a degrading sexual gesture toward you?”) of the Interpersonal Sexual Objectification Scale (ISOS: Kozee et al., 2007). In addition, one item from each of the subscales of the Sexual Experiences Questionnaire (SEQ: Fitzgerald et al., 1999) were used (5 items, e.g., “have you ever been in a situation where a supervisor or co-worker attempted to establish a romantic sexual relationship with you despite your attempts to discourage them”). In Study 1, an exploratory factor analysis (using a promax oblique rotation, Bartlett’s *χ*^2^ = 2894.66, *p* < 0.001) indicated that the items loaded on a single factor (factor 1 loading sum squared of 4.66), so the 8 items were included in a composite measure of previous experience with gendered discrimination (*α* = 0.91). In Study 2, the same measures as in the Study 1 were used but, to distinguish between direct and indirect experiences, we asked participants whether the statements either applied to self or a person close to them. The resulting two scales had acceptable reliability (3 items each, *α* = 0.746 for the direct experiences scale and *α* = 0.824 for the indirect experiences scale).

#### Empathy

The Interpersonal Reactivity Index (IRI: Davis, 1980), a validated and reliable measure of empathy measuring empathic concern and perspective taking (Chrysikou and Thompson, 2016) was used. Two of the four IRI subsets were utilized to measure *empathic concern* (emotional empathy) and *perspective taking* (cognitive empathy). To measure empathic concern six items were used (e.g., “I often have tender, concerned feelings about people less fortunate than me”). In Study 1, seven items were used to measure perspective taking (e.g., “I sometimes find it difficult to see things from the other person’s point of view” reverse coded). Items were averaged to yield an index of *empathic concern* (*α* = 0.774) and *perspective taking* (*α* = 0.803). In Study 2, empathic concern subscale included 6 items (*α* = 0.621). For perspective taking, the assessment of Cronbach’s alpha indicated that the removal of one of the items (“If I’m sure I’m right about something, I do not waste much time listening to other people’s arguments”) would significantly increase the reliability of the scale (from *α* = 0.637 to *α* = 0.751), and as such, this item was not included in the composite sub-scale.

#### Intragroup dissent (Study 2 only)

Three items were used to capture group members’ attitudes in relation to the objectives of the group (i.e., “I think that lately, many #MeToo supporters lost sight of the most important objectives of our movement”), core beliefs (i.e., “Overall, the #MeToo movement does not fully represent anymore my beliefs about addressing the issue of gender inequality in society”), and strategies to reach the group’s goals (i.e., “The means used by the #MeToo movement to reach their objectives are not always the best”). These items were used to create a composite scale for intragroup dissent (*α* = 0.684).

## Results

### Preliminary analyses

Descriptive statistics for the main variables in both studies are shown in Table 1.

**Table 1:** Descriptive statistics (means and standard deviations) for the variables in both studies.

Measured variables	S1: Mean (*SD*)	S2: Mean (*SD*)
Previous experiences (1–7)		
Sexual harassment (direct)	3.304 (1.590)	2.230 (1.356)
Sexual harassment (indirect)	n/a	2.910 (1.692)
Empathy (S1:1–5, S2:1–7)		
Empathic concern	3.917 (0.763)	5.486 (1.023)
Perspective taking	3.778 (0.714)	5.395 (0.967)
Political engagement (1–7)	5.223 (1.314)	3.0734 (1.328)
Group consciousness (1–7)		
Social identification	4.835 (1.163)	4.601 (1.400)
Collective efficacy beliefs	5.530 (1.037)	5.537 (1.482)
Anger at the opposing group	4.235 (1.654)	4.851 (1.717)
Contempt for the opposing group	2.843 (1.638)	3.687 (1.809)
Anger at perpetrators	n/a	6.218 (1.317)
Contempt for perpetrators	n/a	4.814 (1.582)
Behavioral endorsement (1–7)		
Pro-social change action	4.364 (1.509)	4.819 (1.417)
Pro-retribution action	3.818 (1.457)	3.666 (1.460)
Intragroup dissent		
Social change support	n/a	5.88 (1.342)
Retribution support	n/a	5.90 (1.461)
Misalignment	n/a	3.785 (1.250)

Numbers in parentheses next to measured variables in headings column indicate the measurement scale. The measurement scale for empathy differs between Study 1 (1–5) and Study 2 (1–7).

To determine the groups of supporters based on their preference for pro-social change action over pro-retribution and *vice-versa*, we created a variable that would reflect the difference between levels of support of the two types of behaviors (i.e., “diff_support” variable = “endorsement of pro-social change behavior” variable – “endorsement of pro-retribution behavior” variable). This variable was used to categorize the participants in two groups: pro-social change group (i.e., consisting of the participants with scores over 0; *N_*Study1 = 228, and *N_*Study2 = 101) and a pro-retribution group (i.e., consisting of the participants with scores under 0; *N*_Study1 = 128, and *N*_Study2 = 59). Most of our analyses are based on the merged data from both samples; however, we also report the results from Study 2 where we used additional measures.

For both studies we conducted *post hoc* power analyses to determine their statistical power using the software G* Power (Faul et al., 2007, 2009). In both studies, the power analysis was informed by an overall large observed effect size (Cohen’s *d* > 1.05) between the two independent groups. The power analysis was conducted using an alpha level of 0.05, based on the effect size and sample sizes. The analysis shows both studies had an estimated power of over 99% to detect the large effect size noted between the two group means. These findings suggest that the significant differences observed between the two groups are highly likely to reflect a true effect.

### Main analyses

#### Collective dimensions (H1)

We predicted that those supporters mostly endorsing pro-social change action over pro-retribution action will differ in their group consciousness dimensions from those mostly endorsing pro-retribution action over pro-social change action (i.e., pro-social change action supporters will be higher in group identification, anger, and group efficacy than pro-retribution action supporters, who are expected to be higher in contempt). To test this hypothesis, we compared the two groups across the samples. As shown in Table 2, we found that group identification, group efficacy, and anger at the outgroup were significantly higher in the pro-social change group than in the pro-retribution groups. There were no significant differences between the groups in levels of contempt toward the outgroup.

**Table 2:** Means, standard deviations, and *t*-test results in the pro-social change (*N* = 329) and pro-retribution (*N* = 160) groups.

Measure	Pro-social change		Pro-retribution		*t*-test	Cohen’s *d*
	*M*	*SD*	*M*	*SD*		
Identification	4.95	1.17	4.40	1.15	4.74^***^	1.20
Group efficacy	5.62	1.15	5.33	1.16	2.64^**^	1.15
Anger at outgroup	4.57	1.67	4.02	1.67	3.45^***^	1.67
Contempt at outgroup	3.13	1.78	2.90	1.59	1.41	1.72
Empathic concern	4.43	1.09	4.13	1.00	2.95^**^	1.06
Perspective taking	4.31	1.06	3.94	0.99	3.71^***^	1.04
Political engagement	4.68	1.73	4.64	1.63	1.20	1.71
Sexual harassment	3.21	1.62	3.16	1.62	0.311	1.62

^**^p < 0.01, ^***^p < 0.001.

#### Individual dimensions (H2)

We expected that supporters endorsing pro-social change action will differ in their motivations for joining the movement (antecedents of group consciousness) from those endorsing pro-retribution action (to be higher in political engagement and lower in previous experiences of sexual harassment than the second group). Again, we conducted *t*-tests to identify differences between these dimensions. As Table 2 shows, we found significant differences in empathic concern and perspective taking, both significantly higher in the pro-social change group. We found no statistically significant difference between group in their levels of political engagement and experiences of sexual harassment.

#### Pathways to addressing gender inequality (H3)

**Figure 2:**
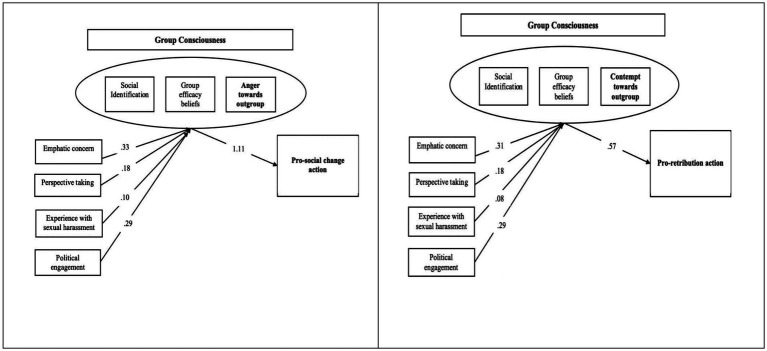
Path models for pro-social change action in the first panel (*CFI* = 0.95; *TLI* = 0.90; *RMSEA* = 0.089) and pro-retribution action in the second panel (*CFI* = 0.90; *TLI* = 0.81; *RMSEA* = 0.118).

We expected that support for pro-social action and support for pro-retribution action will be predicted by different dimensions underpinning group consciousness (pro-social change action should be predicted by a “Group Consciousness” as indicated by identification, group efficacy, and anger, while pro-retribution action would be predicted by a latent variable indicated by identification, group efficacy, and contempt). To test this hypothesis, we conducted SEM analyses where we tested several models. A first model (treating the pro-social change action variable as an outcome) included a latent variable “Group Consciousness” indicated by group identification, group efficacy and anger at the outgroup. A second model (treating the pro-retribution action as an outcome) had the latent variable “Group Consciousness” indicated by group identification, group efficacy and contempt at the outgroup. Both models included the same individual dimensions (life experiences of sexual harassment, empathic concern, perspective taking and political engagement) as antecedents of group consciousness. Figure 2 illustrates these two alternative models, including the path estimates and the model fit coefficients (indicating acceptable to good fit for both models).

## Discussion

The aim of this study was to understand the collective and individual aspects of intragroup fragmentation and its interaction with different forms of action against gender inequality, especially actions that promote social change and actions that promote retribution. We focused on two different categories of supporters within the #MeToo social movement: supporters endorsing a predominantly pro-social change approach (pro-social change action), and supporters endorsing a predominantly retributive approach (pro-retribution action).

Our first hypothesis stated that supporters mostly endorsing pro-social change action would display different dimensions of group consciousness compared to those endorsing pro-retribution action. Specifically, we expected those broadly falling under the categorization of the pro-social change group would score higher in terms of group identification, anger, and group efficacy. Our findings partially supported this hypothesis. While group identification, group efficacy, and anger at the outgroup were significantly higher among the pro-social change group, other dimensions of group consciousness such as contempt at the outgroup did not show significant differences. This suggests that, in line with most research on collective action, the desire for pro-social change (manifested as collective action intentions) is strongly associated with identification with the group, group efficacy, and anger at the injustices perpetrated by the outgroup. However, in our study, the emotion of contempt at the outgroup did not differ between the two groups, indicating that contempt for the outgroup might not be such a relevant collective emotion in this context.

Our second hypothesis focused on the antecedents of group consciousness, assuming differences in motivations for joining the movement. Contrary to our expectations, the data showed no significant differences between the two groups in antecedents of group consciousness such as political engagement and life experiences (experiences of sexual harassment). However, we found that empathic concern and perspective-taking were significantly higher in the pro-social change group. This is a novel finding in the collective action literature, highlighting the importance of diverse individual aspects that can influence our collective selves and associated behaviors. The mixed findings raise questions about the underpinnings of group consciousness and suggest that the motivations for joining such groups may be more complex and multi-faceted than initially thought. Overall, our findings also make sense from a social movement diversification theory perspective (Edwards and McCarthy, 2004) as they suggest that support for various tactics and strategies to achieve the goals of the movement is underpinned by complex and diverse psychological individual profiles, which need to be better understood in conjunction with the collective dimensions explaining participation in social movements.

The third hypothesis sought to explore how different dimensions of group consciousness could predict support for pro-social change action and pro-retribution action. Our SEM analyses presented two alternative models. While the first model linked pro-social change action to a “Group Consciousness” latent variable indicated by identification, group efficacy, and anger, the second model connected pro-retribution action to a latent variable indicated by identification, group efficacy, and contempt. Interestingly, both models included the same individual dimensions as antecedents of group consciousness, suggesting that these underlying factors can drive both pro-social and pro-retribution actions depending on how they interact with group consciousness.

In studying the roles of both the collective and individual aspects of intragroup fragmentation within the #MeToo social movement, the implications of our findings are manifold. First, our findings indicate that while pro-social change supporters scored higher on group identification and anger toward the outgroup, they did not necessarily feel more efficacious as a group. This has implications for social movement participation as well as policy development. It suggests that while fostering group identification and righteous anger can galvanize pro-social change, these alone may not be sufficient for instilling a sense of collective power or efficacy. This is critical for the sustainability and impact of social movements. One possible effective strategy could therefore focus on building opportunities that would not only activate and strengthen identification with the movement and anger toward opponents, but also enhance collective efficacy through tangible wins or educational approaches for example.

An alternative explanation of these findings is that what our data captures is the stage of *diversification* within the process of social movement development (Cunningham et al., 2017; Wang et al., 2019), so there is emerging variance in members support for different types of collective action. However, this aspect of diversification seems to not necessarily translate into greater diversification in terms of the collective dimensions that we included in our study. Future studies should be conducted to determine whether diversification in terms of support for various means to achieve the group goals (i.e., which can be seen as a type of psychological intragroup fragmentation) is manifested later in the process as “real” intragroup fragmentation (i.e., in the form of schism).

Our second hypothesis revealed a more nuanced landscape. We found no significant differences in the “life experience” category of antecedents of group consciousness between pro-social change and pro-retribution supporters, but we did find individual differences in empathic concern and perspective-taking. This suggests that motivations for joining social movements are complex, and the individual traits of the supporters might be particularly important. One implication of our findings is that they imply that these motivations can be harnessed, for example, by building empathy and perspective-taking abilities with the cause. In the context of this study, such approaches could help in converting pro-retribution to pro-social change supporters.

Our models provide a nuanced understanding of how different dimensions underpinning group consciousness can predict different types of actions. This has crucial implications for designing interventions aimed at either promoting pro-social behavior or mitigating retributive actions. For instance, interventions aimed at promoting pro-social change action might need to focus on enhancing group identification, efficacy, and righteous anger. Conversely, if the goal is to reduce retributive actions, interventions may need to address and potentially mitigate levels of group identification, efficacy, and contempt.

On a broader scale, understanding these dynamics can inform public policy and educational programs focused on gender equality. Knowing what drives pro-social and retributive actions can help in the formulation of policies that are not just reactive but also proactive in nurturing a society that values equity and justice.

### Limitations and future research

While this study provides valuable insights into the dynamics of intragroup fragmentation within the #MeToo movement and its implications for pro-social and pro-retribution actions, several limitations must be acknowledged. Firstly, the disparity in sample sizes between the pro-social change group (*N* = 329) and the pro-retribution group (*N* = 160) is not ideal, and future studies should seek to achieve more balanced samples. Also, while the study attempted to consider the role of cultural context, it did not fully explore the impact of various cultural factors that could influence group dynamics and motivations. Future research may need to examine in more depth how cultural factors may influence intragroup fragmentation and different types of actions that movement supporters endorse.

This study is cross-sectional, providing a snapshot of attitudes and behaviors at a single point in time. It cannot account for the dynamic nature of social movements, where attitudes and group dynamics may evolve rapidly. Longitudinal studies are required to understand these temporal changes. The study also relies on self-reported measures, which can be subject to social desirability and other biases. The extent to which these self-reports accurately reflect participants’ true beliefs and actions remains an open question.

Our findings plausibly imply that intragroup fragmentation is more likely to occur in fully fledged, matured social movements where group members have had the time and opportunities to debate and refine identity content including the norms around best achieving the group goals. Increased opportunities for intragroup interactions can also provide group members with opportunities to deliberate and reflect on the collective values, beliefs, norms and aims of the group as a whole. In turn, these can create a platform for disagreement between group members to occur, followed by factionalisation when there dissent about issues which are core to the group identity (Sani and Todman, 2002; Sani, 2008; see also Bliuc and Chidley, 2022). Future research could more explicitly assess this proposition, possibly by using representative samples from populations in different countries and directly test for differences between them. Our findings can also be used as a platform to design follow-up experimental studies that could further investigate the process of intragroup fragmentation in controlled lab conditions. For example, antecedents of intragroup fragmentation and schism such as disagreement on group norms can be experimentally primed to see its effects on intentions to split from the parent group (as per Sani and Todman, 2002).

### Conclusion

Our study contributes to the better understanding of the complex and understudied dynamics of intragroup fragmentation. Our findings highlight the role of group consciousness in driving different forms of action against gender inequality as well as the need for a nuanced understanding of these phenomena, especially in the context of designing interventions aimed at promoting social change. Overall, our research highlights the complexity of the intragroup processes in newly emerging, modern social movements, such as #MeToo. Our analysis suggests that social movements are dynamic, complex, and highly context-dependent, and to understand the processes underpinning their emergence and evolution more accurately, we need extend our investigation to less researched samples and to diverse cultural contexts.

## Data availability statement

The raw data supporting the conclusions of this article will be made available by the authors, without undue reservation.

## Ethics statement

The studies involving humans were approved by Western Sydney University Ethics Committee. The studies were conducted in accordance with the local legislation and institutional requirements. The participants provided their written informed consent to participate in this study.

## Author contributions

A-MB: Conceptualization, Writing – original draft, Writing – review & editing. TH: Data curation, Writing – review & editing. DM: Data curation, Writing – review & editing.
